# Stack-based static WebAssembly binary slicing and mutation for generating valid sub-binaries

**DOI:** 10.1038/s41598-026-45837-y

**Published:** 2026-03-27

**Authors:** GyeongTaek Choi, Seungho Jeon

**Affiliations:** 1 Department of Information Security, School of Cybersecurity, Korea University, Seoul, 02841 Republic of Korea; 2 Department of Smart Security, Gachon University, Seongnam-si, 13120 Republic of Korea

**Keywords:** WebAssembly, Program slicing, Static analysis, Mutation, Computational biology and bioinformatics, Mathematics and computing

## Abstract

WebAssembly is a low-level binary format originally designed to enable high-performance applications to run in web browsers. As WebAssembly is increasingly being ported to various environments, the security verification of WebAssembly execution environments is becoming more critical. While a wide variety of WebAssembly binaries is required to verify these environments, collecting binaries from the wild poses clear limitations. To meet this demand, techniques for automatically generating WebAssembly binaries are necessary. However, automatic generation of WebAssembly binaries must consider not only syntactic validity but also the potential lack of diversity in the generated binaries. To overcome these challenges, this paper proposes a series of algorithms for generating valid and semantically rich sub-binaries from a given base WebAssembly binary. First, closure slices are extracted from the base WebAssembly binary using static program slicing. Then, a stack balance correction algorithm is applied to the closure slices to construct syntactically complete functions. Finally, the generated functions are assembled into a complete WebAssembly binary, and instruction-level mutation is applied to introduce diversity. Several experiments were designed to demonstrate the effectiveness and efficiency of these algorithms, and the evaluation results showed that the proposed algorithms are highly promising in generating sub-binaries.

## Introduction

WebAssembly (WASM)^1^ is a low-level binary format originally designed to enable high-performance applications to run in web browsers. WASM binaries are generated by compiling source code written in high-level languages such as C, C++, or Rust using dedicated compilers like Emscripten^2^. WASM was initially introduced to improve the execution speed of JavaScript-based applications running in web browsers. In fact, despite modern JavaScript engines (V8^3^, SpiderMonkey^4^) employing Just-in-Time Compilation, WASM demonstrates significant performance advantages in real-world applications in terms of execution speed and energy efficiency^5,6^. However, performance differences can vary depending on application type and execution environment. In addition to running WASM binaries within web browsers, they can also be executed via virtual machines such as WASM runtimes^7–10^. Consequently, WASM is now being used not only in web browsers but also in various environments such as cloud computing^11^ and embedded devices^12^.

As WASM continues to be ported to various environments, the security verification of WASM execution environments is becoming increasingly important. In general, verification of WASM runtimes employs techniques such as fuzzing^13,14^ or differential testing^15,16^. These testing methods are known to be effective in uncovering vulnerabilities or bugs in the target systems. However, effective testing of WASM runtimes requires a wide variety of WASM binaries. Collecting WASM binaries from the wild, however, presents clear limitations. Therefore, an automated method is needed to derive diverse binaries from a limited set of existing WASM binaries. Despite the clear demand for WASM binary generation, automatically generating valid WASM binaries faces several challenges. (1) WASM runtimes enforce strict stack-based validation on given WASM binaries. Therefore, the generated WASM binaries must maintain correct stack balance. (2) The instructions that make up a WASM binary have dependencies on various other sections within the binary, such as the global section, table section, and memory section. In other words, for a generated WASM binary to execute correctly, it must be equipped with all necessary execution semantics. (3) Even if a syntactically and semantically valid binary is generated from a given WASM binary, the resulting set of binaries may still lack diversity for execution environment verification. Therefore, strategies to ensure sufficient binary diversity are required. To address the aforementioned challenges point by point, the following insights are adopted: (1) Program slicing^17^ is employed to generate sub-binaries from a given WASM binary. Program slicing is a technique that extracts only the code affecting the behavior at a specific point in the program, producing a reduced subset of the original program. More specifically, program slicing for WASM binaries can be categorized into dynamic slicing, which is performed after observing execution behavior, and static slicing, which does not rely on such observations. Among these, static slicing based on the approach of Stiévenart et al.^18^ is adopted. (2) While Stiévenart et al.^18^ provide a framework for static slicing, their approach replaces a large number of instructions with simple dummy instruction sequences. Instead of this approach, we maintain stack balance by identifying and correcting discrepancies between stack balance and the return types of structured control instructions through multiple static analyzers. (3) Since program slicing merely generates sub-binaries of the base WASM binary, instruction-level mutation is introduced to provide additional semantic richness to the generated binaries. In this paper, a static program slicing method is proposed to generate valid and semantically rich sub-binaries from a given base WASM binary. The slicing approach is fundamentally based on the work of Stiévenart et al.^18^, with a focus on preserving stack balance. More specifically, a function and an instruction within it are randomly selected as slice criteria from the base WASM binary, and then backward slicing is performed to include instructions that are semantically related. A strict static-analysis-based correction algorithm is then applied to ensure that the resulting slice satisfies the required ending stack balance of structured instructions such as block, as well as function type constraints. If the generated slice depends on other functions, the same procedure is recursively applied to those functions. To prevent the generated WASM binaries from becoming unnecessarily large, the function call depth is controlled. Finally, instruction-level stack-preserving backward mutation is applied to maximize the diversity of the generated WASM binary. Several experiments were designed to evaluate the efficiency and effectiveness of the proposed algorithms, and the evaluation results showed that sub-binaries were successfully generated from the base WASM binary with high efficiency. The contributions of this paper are threefold.

- A static program slicing method is proposed for generating sub-binaries from a base WASM binary, along with a static-analysis-based correction algorithm to ensure the validity of the resulting sub-binaries.
- An instruction-level stack-preserving backward mutation technique is introduced to maximize the diversity of the generated sub-binaries.
- A series of experiments are conducted to evaluate the effectiveness and efficiency of the proposed algorithms, through which several limitations and potential solutions are identified.

## Background and related work

### WebAssembly binary

Before discussing program slicing for WebAssembly, it is necessary to understand the structure of a WASM binary. A WASM binary is organized at the module level, and each module consists of 13 sections. Additionally, WebAssembly defines five primitive data types. For integer types, there are i32 and i64, representing 32-bit and 64-bit integers, respectively. For floating-point types, f32 and f64 are defined according to the IEEE 754 standard, depending on their bit width. In addition, a 128-bit vector type called v128 is defined to support high-performance operations. The v128 type is used for single instruction multiple data (SIMD) operations.

**Table 1:** Sections in WASM binary and their descriptions.

Section name	Description
Type section	Define function signatures (parameters and result types)
Import section	Declares external functions, memories, tables, or globals that are imported from other modules
Function section	Declares the types of functions defined in the module
Table section	Describes tables typically used for indirected calls
Memory section	Defines the linear memory that the module uses
Global section	Declares global variables and their initialization values
Export section	Declares which functions, tables, memories, or globals are exported for use by the outside world
Start section	Optional section specifying a function to be called automatically when the module is instantiated
Element section	Used to initialize the table with function indices used for indirected calls
Code section	Contains the function bodies (actual bytecode instructions)
Data section	Defines initial values to write into memory at module instantiation
Data count section	Optional section declaring how many data segments are in the data section
Custom section	Optional section for metadata or debugging information

Table 1 summarizes the main sections that make up a WASM binary and the functions of each section. The type section defines function signatures, specifying the types of parameters a function takes and the type of result it returns. The import section handles the importing of functions, memory, tables, and global variables from external modules, with each item accompanied by type information. The function section lists type indices that indicate which function signatures are referenced by internally defined functions. The table section defines the type of table objects used primarily for indirect calls. Currently, the WebAssembly specification allows only the function reference type, funcref. The memory section specifies the size and constraints of linear memory used by the module, and actual memory access is typically performed based on i32-typed addresses. The global section defines the types and initial values of global variables, which may be shared across the module or used as constants. The export section lists functions, memory, tables, and globals to be exposed to the external environment, forming the module’s interface. The start section specifies an initialization function that is automatically called when the module is instantiated, commonly used for global variable setup or environment initialization. The element section contains information for initializing tables with function indices, thereby enabling indirect calls through a function table. The code section contains the actual implementations of functions, including bytecode instructions for operations, control flow, and memory access. For example, the instruction i32.add pops two i32 operands from the stack, adds them, and pushes the resulting i32 value back onto the stack. The data section includes information for placing initial data into the module’s linear memory and consists of memory offsets and data blocks. The data count section specifies the number of data segments, helping improve the consistency and correctness of the module during static validation. Finally, the custom section is an extensible area that may include name information, debugging metadata, or user-defined content. Although ignored by WebAssembly execution environments, it is useful for static analysis tools and during development.

### Control flow of WebAssembly

WebAssembly supports several structured control instructions (block, loop, and if) and branch instructions (br, br_if, br_table, and return). Structured control instructions bracket sequences of instructions and are terminated by an end instruction. These instructions are implicitly assigned labels, and when nested, the label indices increase by one for each level of nesting. The labels are used in conjunction with branch instructions. The br, br_if, and br_table instructions take a label index as an argument. The br instruction, when encountered, causes an unconditional jump to the structured control instruction associated with the specified label. If the jump target is a block or if instruction, the control flow moves to the end of that instruction. If the target is a loop, the control flow jumps to the beginning of the loop. The br_if instruction checks a condition value from the operand stack. If the condition is true, control flow branches to the structured control instruction associated with the specified label, otherwise it proceeds to the next instruction. Unlike br and br_if, the br_table instruction can take multiple label indices. It pops a selector from the operand stack and uses it to branch to one of the labels defined in the br_table. Lastly, the return instruction immediately terminates the currently executing function and returns the result value. An interesting aspect of WebAssembly’s structured control instructions is that they take into account the stack level at the point of entry. More specifically, when executing the sequence of instructions contained within a structured control instruction and exiting it, the operand stack must match the stack level present at the time of entry. For example, if the operand stack is [i32, f64] at the entry point of a block instruction, this stack state must be preserved upon exiting the block. Additionally, the instructions within the block are not allowed to modify the operand stack below this entry-level depth. Another important point is that WebAssembly’s structured control instructions can have a result type. For instance, if a block instruction has a result type of i32 and the operand stack at entry is [i32, f64], then at the end of the block, the operand stack must be [i32, f64, i32].

**Fig. 1:**
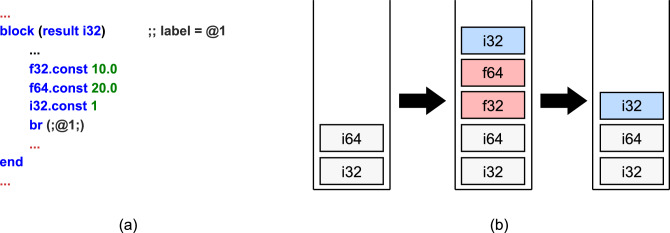
Operand stack unwinding for branching. (**a**) Code snippet. (**b**) Operand stack context for code snippet. Blue represents the return type, and red represents the types to be removed by stack unwinding.

In structured control instructions and branch instructions, an important consideration is stack unwinding. Stack unwinding can occur when a branch instruction causes a jump. Figure 1 illustrates an example of stack unwinding during branching. Figure 1a presents a code snippet of a block instruction with a result type of i32, and Fig. 1b shows the corresponding changes in the operand stack during execution. Upon entering the block, the operand stack is [i32, f64], and the expected stack state upon exiting the block is [i32, f64, i32]. Before the br instruction is executed, f32, f64, and i32 values are pushed onto the operand stack in that order. If control jumps directly to the end of the block in this state, an inconsistency arises because the operand stack no longer matches the expected stack context, which can result in an error. To prevent such errors, WebAssembly performs stack unwinding when a branch instruction is executed. More specifically, among the values pushed onto the stack after the entry-level stack depth, some are saved based on the result type of the target structured control instruction. The values pushed after the stack level are then popped off, and the saved result value(s) are pushed back onto the operand stack. In this way, WebAssembly ensures consistency in the operand stack during branching. As a result, every instruction in a WASM binary maintains exactly one valid stack context, even when there are multiple incoming branches. This property is highly beneficial for static analysis of WASM binaries.

### Related work

Building on the background knowledge presented above, this section introduces research related to the current work. The proposed method is fundamentally based on the work of Stiévenart et al.^18^, who introduced a static, stack-preserving, intra-procedural backward slicing technique for WASM binaries. Their method begins by analyzing data and control dependencies between instructions to compute the set of instructions that affect the slicing criterion, from which a closure slice is constructed. Dummy instructions are then inserted into the closure slice to satisfy WebAssembly’s stack constraints, thereby reconstructing an executable slice. While this study focuses on static analysis-based slicing of WASM binaries, dynamic slicing approaches are also possible. Stiévenart et al.^19^ proposed an execution-based dynamic slicing technique for WASM binaries. This method tracks data and control dependencies at runtime to construct a precise slice that includes only the instructions that actually influence the execution of a given instruction. During the tracing process, the operand stack is faithfully reconstructed, enabling the generation of executable slices that respect the stack-based nature of WebAssembly. Program slicing is not limited to WASM binaries. Umemori et al.^20^ proposed a dependency-cache slicing system at the Java bytecode level. Their approach analyzes control dependencies statically and data dependencies dynamically using an extended Java Virtual Machine (JVM), combining both to compute precise slices. These bytecode slices are mapped back to source code, providing intuitive results to users. Srinivasan et al.^21^ presented an algorithm for precise slicing of x86 32-bit machine code. Their approach operates at the microcode level rather than at the instruction level, decomposing multi-assignment instructions into individual microcode elements for dependency analysis. They also proposed a machine code synthesis technique to reconstruct executable machine code from the sliced microcode. Stoica et al.^22^ introduced a statistical program slicing technique aimed at analyzing the causes of failures in running programs. This method combines hardware-based control flow and memory tracing to approximate dynamic slicing using only runtime-observed information. It provides accurate slices with low overhead and without program modification, making it suitable for error analysis in deployed applications.

## Stack-based static slicing and mutation for WebAssembly binary

In this section, a stack-based static slicing and mutation algorithm is described for generating new WASM binaries from a given base WASM binary. First, an overview of the process by which a new WASM binary is generated from the base binary is presented, followed by a detailed explanation of the algorithm for each step.

**Fig. 2:**
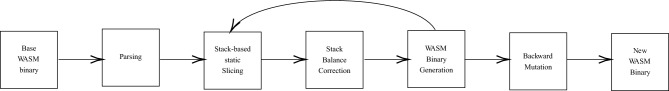
Overview of WASM binary generation.

Figure 2 illustrates the process by which a new WASM binary is generated from a base WASM binary. This process consists of five main steps: (1) Parsing: the base WASM binary is parsed to identify all its sections. During this step, the signatures and instructions of all functions contained in the base binary are extracted. (2) Stack-based static slicing: one of the identified functions is randomly selected as an entry point, and a slice is generated using the static slicing method proposed by Stiévenart et al.^18^. (3) Stack balance correction: although the generated slice reflects control and data dependencies, it does not conform to the syntactic requirements of WebAssembly. To address this, the slice is transformed into a syntactically valid function using a stack balance correction algorithm. (4) WASM binary generation: the generated WASM function may depend on other functions. The process returns to Step 2 to generate the callees in the same way. This loop is repeated to construct a syntactically valid WASM binary. To manage the size of the resulting binary, a method for controlling call depth is introduced. (5) Backward mutation: to maximize the diversity of the syntactically valid WASM binary produced in the previous steps, mutation is applied. This mutation is performed at the function level and proceeds in reverse, taking into account the dependencies between instructions. Once all these steps are successfully executed, a sub-WASM binary derived from the base WASM binary is obtained. It is worth emphasizing that this process incorporates randomness at multiple stages, allowing a completely different sub-WASM binary to be generated each time the process is repeated on the same base binary. The remainder of this section provides a detailed explanation of the algorithms beginning from step 2.

### Stack-based static WebAssembly binary slicing

As previously mentioned, the stack-based static slicing technique proposed by Stiévenart et al.^18^ is fundamentally adopted in this work. Since a portion of that method is reused directly, this paper provides a brief overview of the cited slicing approach and the underlying design rationale. In this stage, after parsing the given base WASM binary, the following operations are performed on a randomly selected function: Stiévenart et al.^18^ generate syntactically valid slices through three main phases: the data-gathering phase, the slicing phase, and the reconstruction phase. Briefly, the data-gathering phase analyzes dependencies among instructions within a function to prepare for slicing. The slicing phase produces a closure slice by traversing instruction dependencies starting from the slicing criteria. Finally, the reconstruction phase ensures the slice is executable by inserting dummy instructions that mimic the stack type effects of the removed instructions, thereby maintaining stack consistency. In this work, the first two phases are adopted as-is, while the reconstruction phase is replaced with the correction algorithm. The rationale for this replacement is that the reconstruction phase in Stiévenart et al.^18^ introduces unnecessary dummy instructions, which reduces the diversity of instructions in the resulting slice. The first step is the data-gathering phase, in which a series of static analyses is performed on the given base WASM binary. Specifically, the following analyses are conducted: stack layout analysis, use-definition chain analysis, memory dependency analysis, global.set instruction analysis, and control dependency analysis. Stack layout analysis calculates the effect each instruction has on the operand stack based on the instruction’s semantics within a function (In this study, the stack layout is referred to as stack balance). Use-definition chain analysis traces the stack behavior of instructions to determine which instruction defines the values used by each instruction. Memory dependency analysis captures data movement through linear memory by identifying load instructions, store instructions, and call instructions (including call_indirect). global.set instruction analysis, similar to memory dependency analysis, reflects data flow through the global section by tracking all global.get and global.set instructions. Control dependency analysis analyzes execution dependencies between instructions to determine which control structures or branching conditions a particular instruction depends on in order to execute. The second step is the slicing phase. The goal of this phase is to produce a closure slice that includes only the instructions necessary for execution, based on the dependencies collected in the previous phase. The process of generating the closure slice is fundamentally designed using a work list algorithm. This algorithm begins by adding the slicing criterion instruction, along with all global.set instructions in the function, to the work list. Then, instructions are iteratively removed from the work list and processed. If the instruction has not yet been included in the slice, it is added, and the algorithm follows its use-definition chains, control dependencies, and memory dependencies to identify related instructions, which are subsequently added back to the work list. Through this iterative process, all instructions that affect the execution of the slicing criterion are included in the slice. Finally, to preserve WebAssembly’s structured control flow, additional branch instructions such as br are inserted where necessary to complete the closure slice.

### Stack balance correction

While the closure slice includes all instructions necessary to execute the slicing criterion, it is not syntactically valid under the WebAssembly specification. As discussed earlier, structured instructions such as block and loop may have result types. However, since the closure slice is constructed in a backward manner starting from the slicing criterion, it often fails to satisfy the required result types of these structured instructions. Furthermore, for the same reason, stack unwinding associated with branch instructions may not be handled correctly in the closure slice. To address these syntactic issues, a stack balance correction algorithm is proposed to ensure the validity of the resulting slice.

**Algorithm 1:**
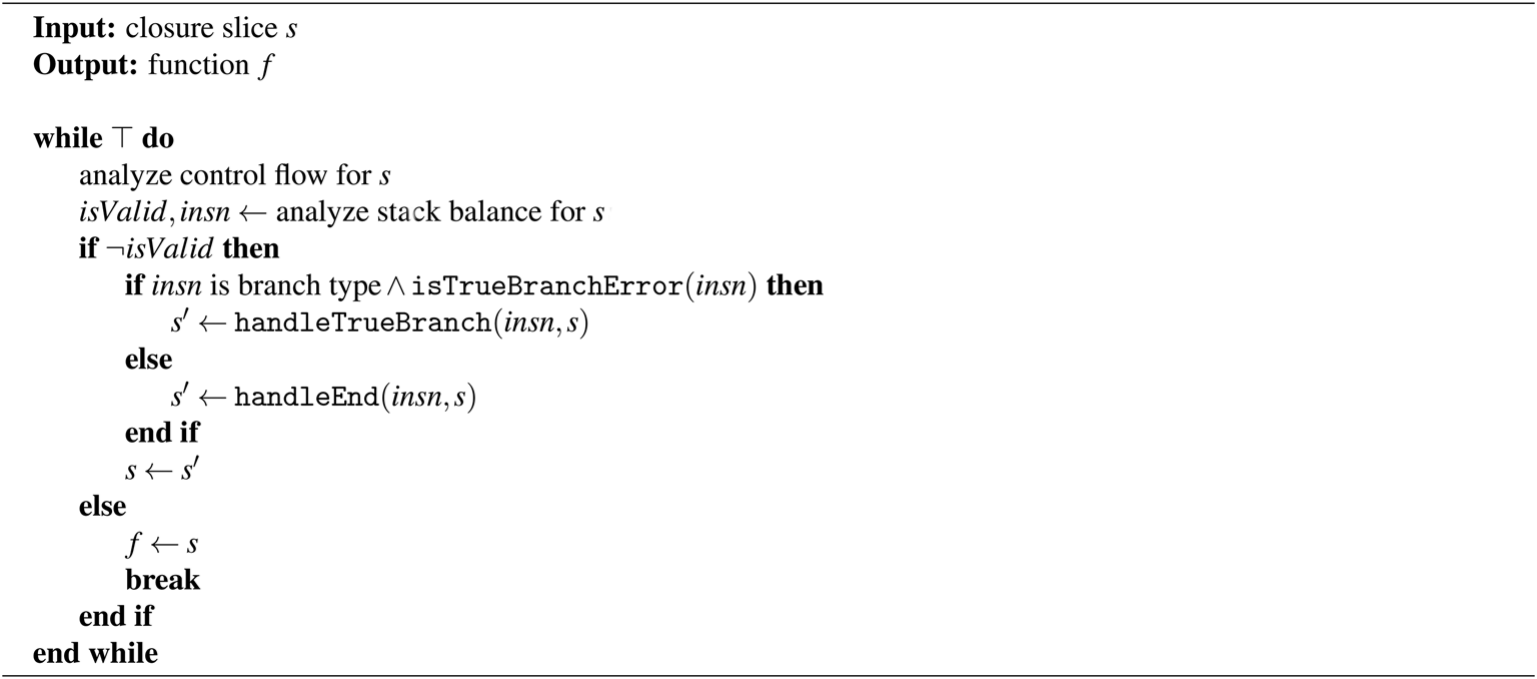
Stack balance correction algorithm.

Algorithm 1 presents the proposed stack balance correction method. This algorithm operates in a straightforward manner. First, a control flow analysis is performed at the instruction level on the input closure slice. Then, based on the control flow, the stack balance of each instruction is analyzed. If any instruction is found to break the valid stack balance, that instruction is identified. According to preliminary experiments, stack imbalance typically occurs at branch instructions (e.g., br) or end instructions in the closure slice. As previously mentioned, this is due to the backward nature of the slicing process. When the identified problematic instruction is a branch instruction and belongs to a true branch-meaning the branch is taken and the execution path actually diverges–the algorithm invokes the procedure handleTrueBranch, which appends additional instructions to the slice to restore stack balance. If, on the other hand, the problematic instruction is an end instruction, the procedure handleEnd is called. The correction process iteratively applies these procedures until all instructions in the slice conform to valid stack balance requirements. The following sections now provide a detailed explanation of the two procedures used in Algorithm 1.

**Algorithm 2:**
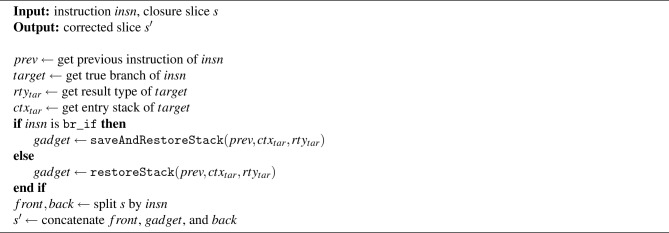
handleTrueBranch procedure.

Algorithm 2 presents the handleTrueBranch procedure. Its goal is to ensure that, before a branch instruction such as br is taken, the operand stack satisfies the stack balance required by the branch *target* (i.e., an end or loop instruction). The algorithm begins by retrieving the instruction *insn* where the issue was detected, its preceding instruction *prev*, and the *target* of the true branch. According to the WebAssembly specification, the *target* of branch instruction can either be an end (in the case of block or if-else) or a loop instruction. It then obtains the *target*’s result type $rty_{tar}$ and the entry stack $ctx_{tar}$. For instance, if the *target* is an end instruction, $ctx_{tar}$ refers to the stack state at the entry point of the corresponding block or if instruction. Using this information, if the instruction *insn* is a br_if, the algorithm calls saveAndRestoreStack to generate a sequence of instructions–referred to as a *gadget*–that adjusts the stack to match the required stack balance. If *insn* is not a br_if, it calls restoreStack instead. The only difference between these two procedures is that saveAndRestoreStack additionally preserves and restores the branch flag that resides on the stack when using br_if. Finally, the algorithm splits the closure slice *s* into two parts–*front* and *back*–around the position of *insn*, and inserts the generated *gadget* between them. The result is a corrected slice $s'$, which restores stack validity at the point of the true branch.

**Algorithm 3:**
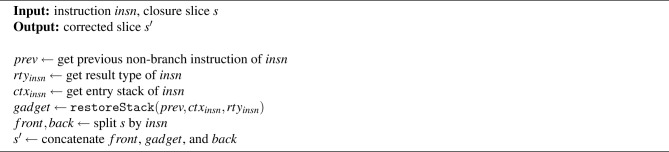
handleEnd procedure.

Algorithm 3 presents the handleEnd procedure. This procedure is fundamentally similar to the previously described handleTrueBranch, with the key distinction that handleEnd assumes the instruction preceding the end instruction is not a branch instruction. The goal here is to ensure that, prior to reaching an end instruction, the operand stack satisfies the stack balance required by the corresponding structured instruction (block or if-else). The algorithm first retrieves the instruction *prev*, which is the instruction executed immediately before the problematic end instruction *insn*. It explicitly excludes cases where the end instruction is reached via a branch instruction. Next, it obtains the result type *rty* of *insn* and the entry stack $ctx_{insn}$, which reflects the operand stack state at the entry point of the corresponding block or if instruction. Using this information, the algorithm calls restoreStack to generate a sequence of instructions–referred to as a *gadget*–that restores the expected stack balance. Finally, the closure slice *s* is split around the position of *insn*, and the *gadget* is concatenated between the two parts to produce the corrected slice $s'$. We now proceed to examine the two core procedures, saveAndRestoreStack and restoreStack, which form the backbone of both handleTrueBranch and handleEnd.

**Algorithm 4:**
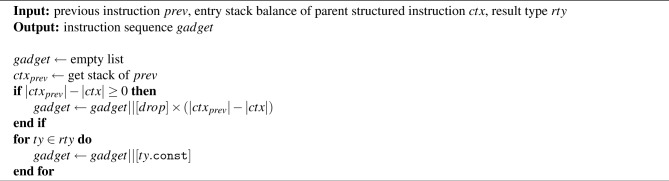
restoreStack procedure.

Algorithm 4 presents the restoreStack procedure. This procedure is designed to be simple and safe. It begins by declaring an empty list, *gadget*, which will store the sequence of instructions needed to restore stack balance. It then retrieves the stack state $ctx_{prev}$ of the previously executed instruction *prev*. If the length of $ctx_{prev}$ exceeds the length of the expected entry stack *ctx*, the difference indicates the number of extra values that must be cleared from the stack. Accordingly, the procedure appends the corresponding number of *drop* instructions to *gadget*. Here, *prev* and *ctx* are derived from Algorithm 2 and Algorithm 3. The underlying assumption is that any values pushed onto the stack after the entry point of the structured instruction and before the problematic instruction *insn* are invalid. Therefore, these values should be removed to restore a clean stack state. Next, the procedure generates the necessary result values to satisfy the result type *rty* of the structured instruction. For each expected result type, it appends a constant-pushing instruction of the form $ty\text {.const}$ to the *gadget*. In this paper, for simplicity, we use $ty\text {.const}$ (e.g., i32.const, f64.const) to push constants directly onto the stack, though instructions such as local.get or global.get could also be used. Since $ty\text {.const}$ embeds the value to be pushed, we initialize it with a randomly generated value appropriate to the type. This procedure thus ensures stack correctness by first cleaning invalid values with drop instructions and then restoring the required result values using type-consistent constants.

**Algorithm 5:**
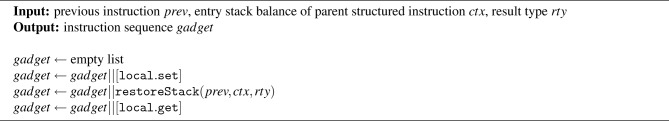
saveAndRestoreStack procedure.

Lastly, Algorithm 5 presents saveAndRestoreStack. This procedure is designed based on the previously described restoreStack. Essentially, saveAndRestoreStack is used to correct stack balance when an issue occurs at the br_if instruction. The br_if instruction is used for conditional branching, and it pops a branch flag from the stack to determine whether to branch. Therefore, saveAndRestoreStack first pops the branch flag from the stack and stores it in a local variable using local.set, then uses the restoreStack procedure to fix the stack balance. Finally, the branch flag stored in the local variable is restored using local.get.

### WebAssembly binary generation with call depth control

Owing to the previous algorithms, a syntactically valid and executable WASM function can be obtained. However, as mentioned earlier, a WASM function may depend on other functions, and multiple sections are semantically required. This section describes how to construct a complete WASM binary using our algorithms.

**Algorithm 6:**
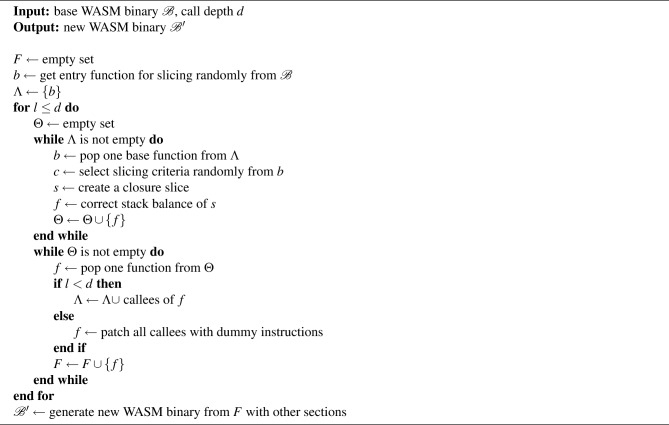
WASM binary generation.

Algorithm 6 presents the proposed method in this paper for generating a WASM binary through call depth control. This process is also designed based on a work list algorithm. It begins by randomly selecting an entry function from the base WASM binary. The selected entry function is added to the work list $\Lambda$. Then, as long as the loop counter *l* is less than or equal to the given call depth *d*, the following operations are repeated. First, an empty set $\Theta$ is declared. $\Theta$ stores the functions generated in each round. While the work list $\Lambda$ is not empty, a base function *b* is taken from $\Lambda$, and slicing and correction are applied to generate a new function *f*. The generated function *f* is included in $\Theta$. After traversing all elements in $\Lambda$, $\Theta$ is traversed. For each function *f* in $\Theta$, if the loop counter *l* is less than the call depth *d*, all callees on which *f* depends are found and appended to the work list $\Lambda$. Otherwise, the callees on which f depends are replaced with dummy instructions. Here, dummy instructions essentially replace the call instruction with a series of drop instructions matching the number of parameters required by the callee, and constant instructions (like *ty*.const in Algorithm 4) are added according to the result type. In the case of the call_indirect instruction, it is sufficient to simply add one additional drop instruction to remove the table index from the stack. Then, *f* is stored in the set of newly generated functions *F*. Once this process is completed, all functions necessary for reconstructing the WASM binary are obtained. Finally, the WASM binary $\mathcal {B}'$ is generated with other sections required for the semantics of the functions in *F*. The reason for designing the algorithm to control call depth is to manage the complexity of the generated WASM binary. By adjusting the size of the call depth, it is possible to generate a WASM binary consisting of a single function or a new WASM binary with the same number of functions as the base WASM binary. Moreover, the generated WASM binary is not only smaller in size compared to the base WASM binary, but also syntactically valid due to the stack balance correction algorithm.

### Instruction-level stack-preserving backward mutation

Finally, a mutation strategy is described to ensure diversity in the generated WASM binaries. As previously mentioned, WebAssembly instructions have designated parameter types and result types. Therefore, the safest and simplest instruction-level mutation that can be considered is to replace an instruction with another one that has the same parameter and result types. While this strategy is valid, it is insufficient for achieving substantial diversity. In contrast, arbitrarily changing the parameter and result types of an instruction and searching for a replacement that matches these types is highly prone to failure. Changing the parameter type of an instruction may require modifying potentially all preceding instructions, while altering the result type may affect all subsequent instructions. Furthermore, due to the relatively limited set of instruction signatures (i.e., combinations of parameter and result types) supported by the WebAssembly specification, arbitrarily chosen types may not correspond to any valid instruction. Therefore, a more cautious mutation strategy is required.

**Algorithm 7:**
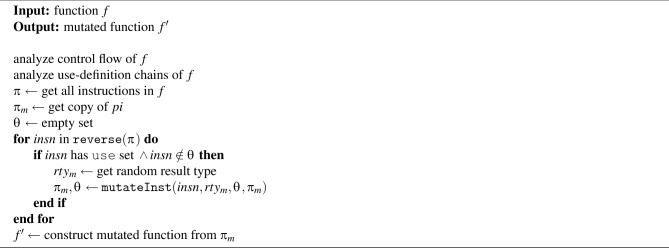
Mutation procedure for function.

Algorithm 7 presents the proposed backward mutation for functions. This algorithm begins with control flow and use-definition analysis for a given function *f*. It is assumed that *f* is included in a generated WASM binary. Then, all instructions $\pi$ of function f and their copy $\pi _{m}$ are obtained. A set $\theta$ is declared to record the mutated instructions. Next, $\pi$ is traversed in reverse order while performing the following steps. If an instruction insn from $\pi$ has a use set (i.e., it requires parameters) and has not yet been mutated, a new result type $rty_{m}$ is randomly selected, and the mutation procedure mutateInst is invoked to mutate the instruction. As described later, mutateInst not only mutates the given instruction *insn* but also mutates all instructions that define its parameters. This process is repeated to update $\pi _{m}$ and mutate all instructions of the given function. Finally, a mutated function $f'$ is reconstructed from $\pi _{m}$.

**Algorithm 8:**
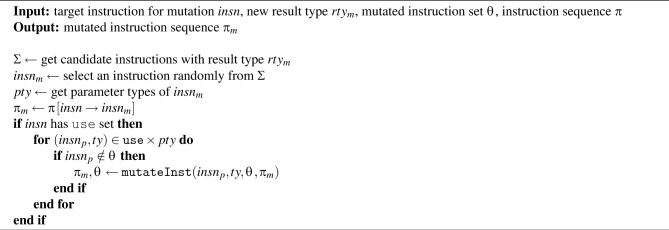
mutateInst procedure.

Algorithm 8 is the mutateInst procedure. This algorithm is designed recursively. The procedure first obtains a set $\Sigma$ of candidate instructions that have the same result type as the given $rty_{m}$. From $\Sigma$, an instruction $insn_{m}$ is randomly selected to replace the original instruction *insn*. Then, the parameter types of $insn_{m}$ are retrieved. The instruction sequence $\pi$ is updated by replacing *insn* with $insn_{m}$, resulting in the mutated instruction sequence $\pi _{m}$. If *insn* has a use set, it is considered to consume operands from the stack. Therefore, each instruction $insn_{p}$ in the use set is recursively mutated so that its result type becomes *ty* (where $ty \in pty$). All functions included in the generated WASM binary are mutated using Algorithms 7 and 8. Through this process, multiple WASM binaries that differ at the instruction level can be obtained. Furthermore, not only can multiple sub-binaries be generated from a single given binary, but diversity in WASM binary generation can also be maximized by continuously using different base WASM binaries.

## Evaluation

This section evaluates the static slicing-based WASM binary generation algorithm. To conduct a thorough evaluation, several research questions (RQs) were established, and a series of experiments were conducted to answer each RQ.

- RQ1. Can the algorithm proposed in this study actually generate syntactically valid WASM binaries?
- RQ2. To what extent is the size of the generated WASM binary reduced compared to the base WASM binary?
- RQ3. How much diversity is achieved in WASM binary generation through mutation?
- RQ4. How time-efficient is the WASM binary generation process?

### Implementation

We implemented the entire process—from parsing the base WASM binary to generating complete sub-binaries—including all algorithms proposed in this study, in 17K lines of Python code (LoC). The implementation adopts a modular architecture with minimal coupling between components, allowing each module to operate independently. The source code is structurally organized. More specifically, the implementation supports all instructions based on the WebAssembly Specification 2.0^23^, and for future research extensibility, each instruction was implemented as a separate class (7K LoC). Parsing of the base WASM binary also follows the approach described in the WebAssembly Specification 2.0. According to the specification, a WASM binary begins with a magic number ($\backslash$ x00 $\backslash$ x61 $\backslash$ x73 $\backslash$ x6d), followed by a version number and sections (see Table 1). We parse all information constituting the base WASM binary and manage it as Python instances. Since the algorithm heavily relies on static analysis, various static analyzers—-including control flow analysis and stack balance analysis—-were also implemented (2.4K LoC). The slicing, stack balance correction, and mutation algorithms were implemented with 150, 550, and 840 LoC, respectively.

### Experimental setup

All experimental evaluations in this section were conducted on a machine with the following specifications: Intel(R) Core(TM) i9-11900 2.50GHz, 32 GB RAM, and 64-bit Ubuntu 20.04 LTS. The proposed algorithm requires a base WASM binary to generate a new WASM binary. To meet this requirement, WasmBench^24^ was adopted. WasmBench contains over 8000 real-world WASM binaries. According to its authors, the dataset includes WASM binaries collected from various code repositories, web applications, and package managers. Finally, to verify the syntactic validity of the generated WASM binaries, the popular WebAssembly toolkit wabt^25^ was used, specifically its wasm-validate tool. wasm-validate is suitable for this evaluation because it not only analyzes the stack balance of a given WASM binary but also reports the exact instruction location where stack integrity is violated.

### RQ1: validity of generated WASM binary

First, the syntactic validity of the WASM binaries generated by the proposed algorithm was evaluated. For this experiment, we randomly selected 1000 binaries from WasmBench with file sizes below the median (approximately 37 KB). The dataset exhibits an approximately normal distribution around the median, making this range representative of typical binaries, and binaries larger than the median are impractical for evaluation due to excessively long parsing and analysis times caused by deeply nested control flow structures. From each base WASM binary, 10 new WASM binaries were derived. Additionally, this configuration is consistently applied across all subsequent experiments. The number of sub-binaries generated per base binary (i.e., 10 in this study) is a configurable hyperparameter of the proposed algorithm, which can be freely adjusted by the user depending on the intended application.

**Fig. 3:**
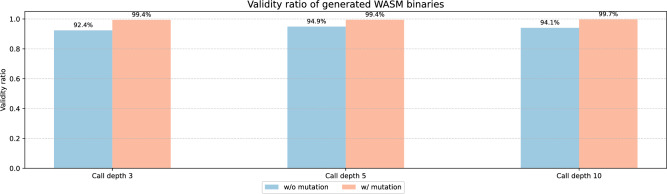
Validity ratio of generated WASM binaries.

Figure 3 shows the validity ratio of the binaries generated by the proposed algorithm as a function of call depth. The validity ratio of the generated WASM binaries was measured by adjusting the call depth parameter. In the figure, the blue bars represent the validity ratio of non-mutated WASM binaries, while the red bars represent that of mutated WASM binaries. As shown in the figure, approximately 92–94% of the WASM binaries were valid when mutation was not enabled. In contrast, about 99% of the binaries were valid when mutation was applied. The incomplete validity was fundamentally attributed to our implementation issues. Through our in-depth analysis, we confirmed that such invalidity arises from mismatches between return types and stack balance at the return points of some structured control instructions. In other words, despite the soundness of our algorithms, this occurs because our implementation does not correctly handle stack unwinding. This can be resolved through further investigation. The higher validity ratio in the presence of mutation is entirely attributed to the implementation design. Specifically, before mutation is applied (i.e., after completing Algorithm 6), all sections except the type, code, and function sections are inherited directly from the base WASM binary. However, after mutation, all sections of the generated WASM binary are carefully adjusted to ensure that the mutated instructions are semantically supported. For example, load and store instructions refer to linear memory through offsets. Therefore, the generated WASM binary must include a memory section, and memory-related information such as data section indices must be adjusted if necessary. As a result, mutated binaries are handled more delicately, leading to a higher validity ratio compared to non-mutated binaries. Another interesting observation is that the validity ratio is not affected by call depth. In other words, the complexity of the generated WASM binary does not compromise the safety of the iterative algorithm.

### RQ2: size reduction

Since the algorithm proposed in this study is fundamentally based on static slicing, the generated WASM binaries are reduced in size compared to the base WASM binaries. This experiment evaluates the extent of size reduction in terms of the number of instructions. The reason for not using file size as a metric is that it is less reliable compared to instruction count. For example, the data section represents pre-defined data located in linear memory. However, this section can be arbitrarily controlled by the user and can significantly affect the overall size of the WASM binary. Therefore, to ensure a fair comparison, the number of instructions is used as the evaluation metric.

**Table 2:** Evaluation for the number of total instructions and reduction ratio in WASM binaries. min/mean/max: the minimum/mean/maximum number of instructions in WASM binaries. base: base WASM binaries. w/o mut and w/ mut: without/with mutation. Percentages indicate the ratio of the generated binary’s instruction count relative to the corresponding statistic (min/mean/max) of the base WASM binaries.

	Min			Mean			Max		
Base	5			6,331			18,343		
	Call depth 3			Call depth 5			Call depth 10		
	Min	Mean	Max	Min	Mean	Max	Min	Mean	Max
w/o mut	2 (40%)	552 (8.71%)	3,869 (21.09%)	3 (60%)	531 (8.38%)	6,561 (35.76%)	3 (60%)	578 (9.12%)	6,628 (36.13%)
w/ mut	2 (40%)	557 (8.79%)	4,182 (22.79%)	3 (60%)	542 (8.56%)	6,701 (36.53%)	3 (60%)	582 (9.09%)	7,062 (38.49%)

Table 2 shows the changes in the total number of instructions contained in WASM binaries generated through the proposed algorithm. The percentage in each cell represents the ratio of the instruction count of the generated binary to the corresponding statistic of the base WASM binaries (i.e., min, mean, or max). Among the WASM binaries used in this experiment, the smallest base binary contained 3 instructions, while the average and maximum were 6,331 and 18,343 instructions, respectively. Using these base WASM binaries, variations in the number of instructions in the generated WASM binaries were analyzed based on call depth and whether mutation was applied. First, when the call depth was set to 3, non-mutated binaries contained a minimum of 2 instructions, an average of 552, and a maximum of 3,869 instructions. In contrast, mutated binaries contained a minimum of 2, an average of 557, and a maximum of 4,182 instructions. This confirms that the generated binaries generally include far fewer instructions compared to the base WASM binaries. Next, regarding the impact of call depth, it was observed that increasing call depth in binary generation generally resulted in WASM binaries with more instructions. For example, while non-mutated binaries at call depth 3 contained up to 3,869 instructions, those generated at call depth 10 contained up to 6,628 instructions. Additionally, mutated binaries tended to contain more instructions than their non-mutated counterparts. This increase is attributed to the additional instructions introduced during post-mutation stack balance correction, which ensures the syntactic validity of the mutated WASM binaries.

**Table 3:** Evaluation for the number of instructions and reduction ratio per function. The column names are same with those in Table 2. Percentages indicate the ratio of the generated function’s instruction count relative to the corresponding statistic (min/mean/max) of the base WASM binary functions.

	Min			Mean			Max		
Base	1			144			4,263		
	Call depth 3			Call depth 5			Call depth 10		
	Min	Mean	Max	Min	Mean	Max	Min	Mean	Max
w/o mut	1 (100%)	53 (36.8%)	458 (10.74%)	2 (200%)	51 (35.41%)	600 (14.07%)	3 (300%)	52 (36.11%)	680 (15.95%)
w/ mut	1 (100%)	53 (36.8%)	472 (11.07%)	2 (200%)	52 (36.11%)	614 (14.4%)	3 (300%)	52 (36.11%)	683 (16.02%)

Table 3 presents the average number of instructions per function. The percentage in each cell represents the ratio of the instruction count per function in the generated binary to the corresponding statistic of the base WASM binary functions (i.e., min, mean, or max). The experimental settings are identical to those used in Table 2. As with the results in Table 2, the functions in the generated WASM binaries contained, on average, fewer instructions compared to those in the base WASM binaries. In addition, the generated binaries showed a trend of increasing instructions per function as the call depth increased or when mutation was applied. This reflects that higher call depth leads to more complex function compositions, and mutation introduces additional instructions—especially due to stack balance correction–resulting in an increase in the average instruction count per function. In conclusion, when mutation was enabled, the generated WASM binaries contained between 22% (at call depth 3) and 38% (at call depth 10) of the maximum number of instructions compared to the base WASM binaries. Additionally, the average number of instructions per function ranged from 11% (at call depth 3) to 16% (at call depth 10) of the maximum instruction count per function in the base WASM binaries.

### RQ3: diversity of generated WASM binaries

Thus far, it has been confirmed that the algorithm proposed in this study can generate valid WASM binaries while effectively reducing their size. In this evaluation, the focus shifts to examining how much diversity the proposed mutation strategy can introduce in the generation of new WASM binaries.

**Fig. 4:**
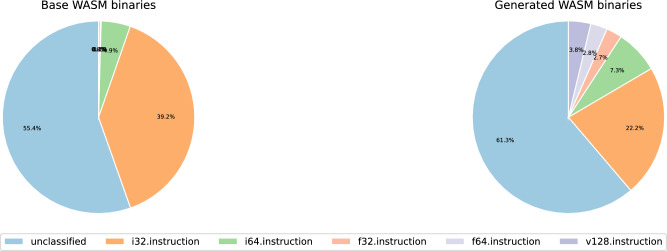
Instruction distribution of base and generated WASM binaries according to data type.

Figure 4 illustrates the distribution of instruction types in both the base WASM binaries and the generated WASM binaries. It is important to note that the WebAssembly specification does not explicitly categorize instructions by type. Instead, in this study, instructions were classified based on the type of value they produce. For example, i32.add consumes two i32 values from the stack and pushes back one i32 value, and is therefore classified as an i32.instruction. Similarly, i64.instruction, f32.instruction, f64.instruction, and v128.instruction are categorized using the same criterion. The unclassified category includes instructions that do not produce any values, such as branch instructions and structured instructions like block. As shown in the figure, excluding unclassified instructions, the base WASM binaries predominantly consist of i32.instruction and i64.instruction. This trend is not unique to WebAssembly but is common across most types of programs, due to the prevalence of integer operations. For example, most programming languages use loops controlled by integer index variables, and references and dereferences to memory rely on addresses, which are typically represented as 8-byte pointers on modern 64-bit machines. Regardless of the reason, such base WASM binaries exhibit limited diversity in instruction types. In contrast, the WASM binaries generated by the proposed algorithm display a more balanced distribution across instruction types. While unclassified and i32.instruction categories still account for the largest proportions, the presence of other instruction types has noticeably increased. In particular, v128.instruction, which is rarely observed in base WASM binaries, shows a significant rise in the generated binaries. This demonstrates that the proposed algorithm introduces sufficient diversity in the generation of WASM binaries.

**Fig. 5:**
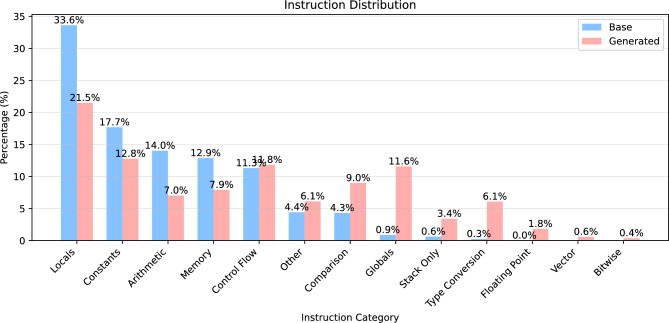
Instruction distribution of base and generated WASM binaries according to functional category.

Figure 5 presents the comparison of the instruction distribution of base WASM binaries and generated WASM binaries by functional category. Where functional categories classify instructions according to their functional role and purpose. We defined 13 categories: Locals, Globals, Memory, Control flow, Constants, Comparison, Arithmetic, Bitwise, Type conversion, Floating point, Stack only, Vector, and Other. Locals are instructions that read or write local variable values. Globals are instructions that read or write global variable values. Memory are instructions that load/store data from linear memory or manage memory size. Control flow are instructions that control program execution flow. Constants are instructions that push constant values onto the stack. Comparison are instructions that compare two values to determine conditions. Arithmetic are instructions that perform arithmetic operations. Bitwise are instructions that perform bitwise operations. Type conversion are instructions that perform conversions between different data types. Floating point are instructions that perform floating-point operations. Stack only are instructions that only manipulate the stack. Reference are instructions that handle reference types and tables. Vector are instructions that perform SIMD vector operations. Atomic are instructions that perform atomic memory operations. Finally, Other are instructions that do not belong to the above categories. Base binaries show a highly skewed distribution, with 78.2% of all instructions concentrated in the top four categories (Locals 33.6%, Constants 17.7%, Arithmetic 14.0%, Memory 12.9%). In particular, advanced operation instructions such as Floating Point, Vector, and Bitwise are nearly absent or completely missing. In contrast, generated binaries show a more balanced distribution across multiple categories, with even the top category, Locals, accounting for only 21.5%, and including Other (11.8%), Constants (12.8%), and Control Flow (11.6%). More notably, Vector instructions, which were 0.0% in base binaries, appear at 1.8% in generated binaries, and significant increases are observed in categories such as Comparison (4.3%$\rightarrow$9.0%), Globals (0.9%$\rightarrow$6.1%), Type Conversion (0.3%$\rightarrow$6.1%), and Stack Only (0.6%$\rightarrow$3.4%). These results clearly demonstrate that our mutation-based approach effectively enhances instruction diversity not only at the type level but also at the functional level, and can provide much more comprehensive coverage than base binaries for testing various implementation paths of WASM runtimes. In contrast, Stiévenart et al.^18^, the direct ancestor of this work, fundamentally uses dummy instruction sequences composed of predetermined instruction types to correct closure slices with broken stack balance. More specifically, if the closure’s operand stack is larger than that of the original binary, a series of drop instructions are added, and if the closure’s operand stack is smaller, t.const instructions are added. This clearly does not contribute significantly to instruction diversity in sub-binaries. We attempted to reproduce the implementation of Stiévenart et al.^18^ to demonstrate this, but encountered issues that suggest their implementation does not fully support the WebAssembly specification. Therefore, we present this analysis instead of a comparative evaluation.

**Table 4:** Unique instruction ratio according to the settings.

Setting	# total instructions	# unique instructions	Unique instruction ratio
Base binaries	9737956	348	$3.57 \times 10^{-5}$
Generated w/o m	462837	298	$3.21 \times 10^{-4}$
Generated w/ m	369015	842	$1.14 \times 10^{-3}$

Table 4 compares the total number of instructions and unique instructions in WebAssembly binaries across different settings. We randomly sampled 1000 base binaries from the dataset and generated 1,000 binaries without mutation and 1,000 binaries with mutation applied from these base binaries. To further assess the diversity of generated binaries, we measured the unique instruction ratio across three settings: base binaries, generated binaries without mutation, and generated binaries with mutation. Table 4 presents the results from 1000 binaries in each setting. The base binaries contain 348 unique instructions out of 9,737,956 total instructions, yielding a ratio of $3.57 \times 10^{-1}$. Generated binaries without mutation exhibit 298 unique instructions from 462,837 total instructions, resulting in a ratio of $3.21 \times 10^{-4}$, approximately 9 times higher than the base binaries. This increase is primarily attributable to the slicing process, which reduces total instruction count while maintaining a relatively concentrated set of unique instructions. Notably, the application of mutation dramatically enhances diversity, producing 842 unique instructions from 369,015 total instructions with a ratio of $1.14 \times 10^{-3}$. This represents a 32-fold increase compared to base binaries and demonstrates that mutation effectively introduces substantially more varied instruction patterns. The significant expansion in unique instruction coverage is particularly valuable for runtime verification and security testing, as it exercises a broader range of instruction decoder paths and edge cases within WebAssembly runtime implementations.

### RQ4: time efficiency for WASM binary generation

Finally, the time required by the proposed algorithm to derive new WASM binaries from base WASM binaries was evaluated. Time efficiency is a critical factor. The primary motivation for generating new WASM binaries is to enable pinpoint analysis of specific behaviors in base binaries or to verify implementation issues in WASM runtimes. If generating a single WASM binary takes an unacceptably long time, it would hinder the practical use of the algorithm for such purposes.

**Fig. 6:**
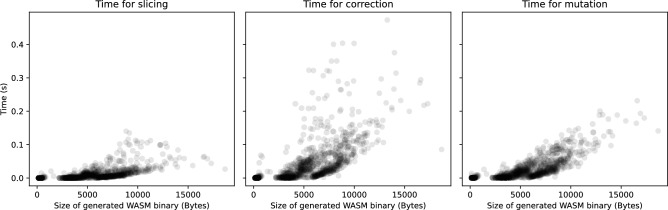
Time costs for slicing (left), correction (center), and mutation (right) steps according to the size of generated WASM binaries.

Figure 6 shows the time taken for slicing, correction, and mutation relative to the size of the generated WASM binaries. Note that the reported time does not include the time for parsing the base WASM binaries or writing the generated binaries to disk. As illustrated in the figure, all three stages exhibit a moderate exponential growth in time. In other words, as the size of the generated WASM binary increases, the time complexity tends to grow rapidly. Nevertheless, even when the size of the WASM binary reaches 15KB, the time taken for slicing, correction, and mutation remains under one second in all cases. Among the three, slicing took the least amount of time, followed by mutation, while correction consumed the most. The relatively high time cost of stack balance correction is due to the repeated execution of computationally expensive static analyses needed to fix syntactically invalid closure slices. Similarly, the lower time efficiency of the mutation phase compared to slicing can be attributed to the recursive and backward nature of the mutation algorithm, which not only modifies individual instructions but also recursively adjusts dependent instructions. Despite these characteristics, all three algorithms demonstrate acceptable levels of time efficiency, making them suitable for practical use.

### Comparative study with baseline slicing methods

Despite the comprehensive evaluation presented earlier, we conduct a comparative study with baseline approaches to objectively assess the proposed algorithms. To the best of our knowledge, there are only a few existing studies regarding WebAssembly binary slicing. We selected Stiévenart et al.^18^ and Stiévenart et al.^19^ as baselines for comparison.

**Table 5:** Comparative study for our method with baseline slicing methods.

Criteria	Stievenart et al.^18^	Stievenart et al.^19^	Our method
Slicing type	Static,intra-procedural, backward	Dynamic,inter-procedural	Static, backward with call depth control
Diversity mechanism	Uses only fixed dummy instructions (drop, const)	No diversity mechanism implemented	Implements instruction-level backward mutation with random type selection
Output generation	Generates single intra-procedural function slices only	Extracts and slices individual functions without call depth control	Generates complete binaries with configurable call depth control
Stack balance	Inserts mass dummy instructions via reconstruction phase	Validates stack per deletion through ORBS execution	Applies iterative correction using strict static analysis with context-aware gadgets
Validity assurance	Stack specification analysis with reconstruction achieves 99.991% success	Execution-based validation per deletion guarantees 100% executable slices	Iterative correction without mutation achieves 92-94%, with mutation reaches 99%+
Specification compliance	Partial WASM 1.0 implementation with MiniWasm subset	ORBS framework with line-level deletion on 57 C programs	Near-complete WASM 2.0 including full SIMD support

While quantitative comparison through direct experimental reproduction would be ideal, we encountered practical difficulties in executing the publicly available implementations of the baseline approaches. These difficulties appear to stem from incomplete WebAssembly specification coverage in the existing implementations, which limits their applicability to the diverse set of real-world binaries used in our evaluation. Consequently, we conduct a qualitative comparison based on the methodologies, capabilities, and evaluation results reported in the original publications. Table 5 presents a qualitative comparison of three WebAssembly binary slicing approaches across six key criteria. Stiévenart et al.^18^ implement a static, intra-procedural slicing method that achieves high performance (0.67s average) and validity (99.991%) but produces relatively large slices (53% of original size) using fixed dummy instructions for stack balance. Stiévenart et al.^19^ employ dynamic observational-based slicing (ORBS)-based slicing that generates the smallest slices (13.17%) with guaranteed executability through per-deletion validation, though at substantial time cost (11.1 minutes average). Both approaches lack explicit diversity mechanisms and focus on single-function or extracted-function slicing without complete binary generation capabilities. The proposed manuscript distinguishes itself through three key contributions. First, an instruction-level backward mutation mechanism that provides diversity essential for security testing applications, second, complete binary generation with configurable call depth control enabling flexible output from single functions to multi-function binaries with all necessary sections, and third, near-complete WebAssembly 2.0 implementation with full SIMD support. The approach achieves fast execution (<1 s for binaries under 15 KB) with reasonable slice sizes (22–38%) and demonstrates that mutation significantly improves validity from 92 to 94% to over 99%. While existing approaches target program comprehension and reverse engineering, the manuscript specifically addresses runtime verification, fuzzing, and differential testing scenarios where diverse, complete, and valid binaries are essential requirements.

### Case study with more realistic WASM binary

In this section, we validate the effectiveness of our proposal using more realistic WASM binaries than those in WasmBench. For this case study, we adopt a binary implementing the Sobel filter from the WebAssembly examples^26^ provided by Mozilla Developer Network (MDN). The Sobel filter is one of the popular image filters used for edge detection in images.

**Table 6:**
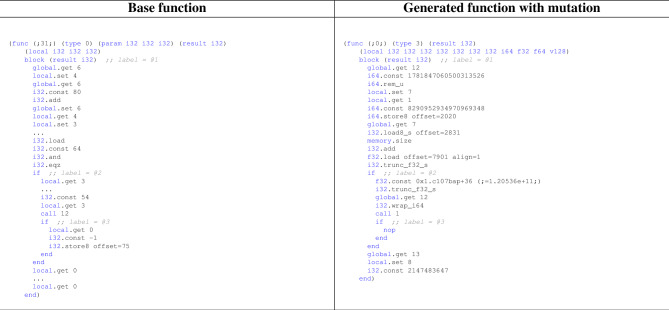
Comparison between base function and generated function with mutation.

First, the Sobel filter binary consists of 9 Type sections, 33 Functions, 1 Table section, 1 Memory section, 6 Global sections, 1 Element section, 6 Data sections, 22 Import sections, and 20 Export sections. From this, we successfully generated a binary with 9 Type sections, 10 Functions, 1 Table section, 1 Memory section, 16 Global sections, 1 Element section, 1 Data section, 0 Import sections, and 10 Export sections. Table 6 compares a randomly selected function from the base WASM binary (left side) with the function generated from it (right side). For reference, the base function contains 50 instructions, while the derived generated function has 28 instructions. Moreover, as shown in Table 6, the base function is fundamentally monotonous, consisting only of i32-type instructions. In contrast, the generated function is not only reduced in size through program slicing but also contains a well-balanced mix of various instruction types. This reconfirms what we observed in Fig. 4: binaries generated by our method exhibit diverse instruction distributions. Furthermore, the generated function fundamentally preserves the control flow of the base function. This is because control-dependency analysis in program slicing includes all instructions that affect the control flow until reaching the slicing criteria.

## Discussion

In this section, the static slicing-based WASM binary generation and mutation algorithms proposed in this study are discussed. Several research questions were established, and a series of thorough experiments were conducted to demonstrate the efficiency and effectiveness of the proposed algorithms. Despite the comprehensive evaluation, several limitations have been identified.

- This study begins with the generation of closure slices as proposed by Stiévenart et al.^18^, which relies on several static analyses such as control flow analysis and use-definition chain analysis. Among these, the time efficiency of closure slice generation heavily depends on control dependency analysis. Currently, this analysis is based on an iterative post-dominator algorithm^27^, which becomes less efficient as the complexity of the function graph increases. To overcome this limitation, more efficient algorithms such as the Lengauer-Tarjan algorithm^28^ could be introduced.
- The correction algorithm proposed in this study identifies instructions that break stack balance within closure slices and computes the instructions required to restore the stack. However, this process involves repeated execution of relatively expensive static analyses, including control flow analysis and stack balance analysis. This inefficiency stems from the algorithm’s design itself. In the short term, optimizing the static analysis algorithms could mitigate this issue, while in the long term, research into fundamentally more efficient algorithms is necessary.
- The mutation algorithm presented in this paper fundamentally works at the instruction level. While this is a fine-grained approach to providing diversity in WASM sub-binaries, there could be several other possible mutation approaches. For example, control-flow-level mutation can be considered. The structured instructions in the WebAssembly specification, such as block or if instructions, fundamentally only concern stack balance and return type. In other words, it is acceptable to replace a block instruction with an if instruction. However, special features depending on the instruction type–such as the conditional flag of the if instruction or the branching condition of the loop instruction–must be taken into account. Beyond control-flow-level mutation, various other mutations can be considered, such as crossover between functions with matching signatures across multiple binaries.
- The experiments in this study were conducted using only binaries below the median file size in WasmBench. Although this was sufficient to observe the complexity trends of the proposed algorithms, evaluating the full dataset would have provided stronger experimental validity. Furthermore, while the proposed algorithms already generate valid sub-binaries at a high rate, the validity is not yet complete. As discussed in RQ1, the remaining invalidity stems from implementation issues related to stack unwinding, which must be resolved to ensure the full correctness of the proposed approach.

Despite the aforementioned limitations, the benefits of our proposed WASM binary generation method are clear. As previously mentioned, this study can be utilized to identify security vulnerabilities in WASM runtimes. Differential testing is widely adopted to identify security vulnerabilities in WASM runtimes^15,16^. This approach executes multiple WASM runtimes with a single WASM binary and observes differences in their behaviors. If a WASM runtime exhibits behavior different from other runtimes, this indicates that it does not correctly support the WebAssembly specification, which can potentially lead to vulnerabilities. While differential testing is highly effective for verifying WASM runtimes, it requires a substantial amount of WASM binaries. In particular, WASM binaries for such verification must be sufficiently small to enable fast and iterative testing of WASM runtimes while possessing diversity to ensure adequate coverage of WASM runtime implementations. To meet this requirement, the research and industry communities have been proposing methods to automatically generate diverse WASM binaries, and our research presents a novel method that satisfies such requirements. Beyond this, we expect that our research can be utilized in various other areas, such as regression testing for WASM runtimes and compilers.

## Conclusion

In this paper, static slicing, correction, and mutation algorithms were proposed to generate diverse WASM binaries. The slicing algorithm is fundamentally based on the work of Stiévenart et al.^18^. However, that approach uses only a fixed set of instructions to repair the stack balance of the generated closure slices, resulting in limited diversity in the generated WASM binaries. To address this, a new stack balance correction algorithm was introduced. This algorithm leverages several static analyses to iteratively identify instructions that break stack balance and randomly selects appropriate instructions to restore it, thereby enhancing diversity. In addition, an instruction-level backward mutation algorithm was proposed to further maximize the diversity of WASM binaries. The proposed algorithms were thoroughly evaluated through a series of experiments, which demonstrated their benefits while also revealing several limitations. The causes of these limitations and potential ways to overcome them were discussed, which will serve as directions for future work. In addition, verification of the WebAssembly execution environment based on our WASM binary generation technique will also be included in the scope of follow-up research.

## Data Availability

The datasets analysed during the current study are publicly available. The base WebAssembly binaries used in the experiments were obtained from WasmBench, which is accessible at https://github.com/sola-st/WasmBench.

## Code availability

The custom code developed in this study is publicly available on GitHub at https://github.com/ssa-lab-gachon-Univ/wasm-slice. The repository contains all implementations of the proposed algorithms, including the static slicing, stack balance correction, and mutation procedures described in this paper.
